# The public you want, the public you get: Exploring the relationship between the public and science in the debate on xenotransplantation

**DOI:** 10.1177/09636625241232098

**Published:** 2024-03-04

**Authors:** Johannes Kögel

**Affiliations:** Ludwig Maximilian University of Munich, Germany

**Keywords:** biopolitics, biotechnology, citizen participation, functional differentiation, unmediated public, xenotransplantation

## Abstract

The debate that followed the first-in-human cardiac transplantation of a genetically modified pig organ emerged as a discussion of social justice when the patient’s criminal record was revealed. This article aims to make sense of this debate by understanding the role of the ‘public’ today, particularly in relation to the governance of biotechnology. The relationship between the public and science is increasingly mediated through citizen participation. However, the public debate that unfolded on matters of social justice can be seen as an unmediated public discourse, which carries the risk of producing unpredictable outcomes. The content of the debate gains significance due to the functional differentiation of society. The medical subsystem does not consider the patient’s history in terms of their involvement in the legal sphere, that is, their criminal record. Nevertheless, normative judgements are transferred across functional systems, allowing for the influence of public opinion and the potential for public scorn.

## 1. Introduction

On 7 January 2022, the world witnessed the first-ever transplantation of a transgenic pig heart into a human patient at the School of Medicine of the University of Maryland (Kotz, 2022; Rabin, 2022a). This novel procedure, known as xenotransplantation, involves the transplantation of organs, cells or tissues across species, as opposed to allotransplantation, which refers to transplantation within the same species. It is important to note that this transplantation was not part of a clinical study but rather a compassionate use case, authorized by the Food and Drug Administration (FDA) of the United States under emergency circumstances.

The recipient of the xenograft was David Bennett, a 57-year-old patient who was suffering from a life-threatening arrhythmia. At the time of the transplantation, Bennett was bedridden and dependent on a heart-lung machine (ECMO) for survival. Due to his history of non-compliance with medical standards, he was deemed ineligible for an allotransplant. In addition, his arrhythmia ruled out the possibility of receiving an artificial heart device. However, following the transplantation of the transgenic pig heart, it immediately began functioning without any signs of organ rejection. After a few days, Bennett was able to be disconnected from the ECMO machine. Although he experienced a few episodes of infection, Bennett’s condition remained stable for up to 8 weeks post-transplantation. Unfortunately, his health rapidly deteriorated within a few days, leading to multiorgan failure and he passed away 2 months after the transplantation.

The focus of this article is to explore the relationship between science and the public in the context of xenotransplantation. To begin, I will delve into the debate that emerged shortly after the xenotransplantation took place, when it was publicly revealed that Bennett had a criminal record. Public opinion, as expressed through comments and social media posts, centred around matters of social justice and whether Bennett deserved a second chance in the form of a potentially life-saving xenotransplantation. It is worth noting that the purpose of this article is not to dissect the debate itself or the independent dynamics of public opinion and discourse but rather to provide insight into the current landscape of public engagement with biotechnology.

By comparing the public debates surrounding the first human cardiac transplantation in 1967 and the first cardiac transplantation of a transgenic pig organ in 2022, I will examine the concept of ‘the public’ and how public consultation has evolved to incorporate citizen participation. Furthermore, I will explore why xenotransplantation has emerged as a pioneering field in terms of public engagement. Finally, I will discuss the significance and role of the unmediated public in the ongoing debate about David Bennett.

## 2. Public debates of heart transplantation

### Looking back to 1967

The success of the world’s first heart transplantation performed by Christiaan Barnard in 1967 was partly attributed to its portrayal in the media. Historian Ayesha Nathoo, who examined the media coverage at that time, concludes:

That Barnard’s operation was the world’s first transplantation of the human heart seemed sufficient to mark it as self-evidently ‘historic’, without journalists offering reasons for this claim, and the language and style of the reporting contributed to establishing it as such. (Nathoo, 2009: 60)

During this period, Christiaan Barnard became a superstar, and Louis Washkansky became the world’s most famous patient. Washkansky ‘was portrayed as an ordinary man, an “average” man, someone with whom readers could identify: [. . .] a family man, with soft likeable features and a big smile, being given a last chance of life. He was a unique person, yet also “Everyman”’ (Nathoo, 2009: 67). One significant topic of discussion among journalists at the time revolved around the identity of the heart donor and how Washkansky supposedly assimilated to this circumstance. He was asked about having a ‘female heart’ or, given his Jewish background, having the heart of a Gentile beating in his body (Nathoo, 2009: 71). This highlights the symbolic significance of the heart, which carries identity-defining value beyond being a mere mechanical pump or organ. It also reflects the belief that the organ may carry characteristics of its previous owner, a notion described by Mary Douglas (1976) as ‘half-identity’.^1^

Regarding public response, Nathoo (2009: 63) reconstructs the debate that unfolded in the United Kingdom within the readership of The Sun, focusing on social justice issues and questioning who deserves to be saved and who should have the authority to make such decisions. This discussion occurred without taking into account the prognosis of the procedure at that time or the fact that Washkansky died 18 days after the operation. It led to a debate about whether the patient was a suitable candidate and whether the operation should have been performed in the first place (Nathoo, 2009: 69).

Following the first implementation in South Africa, heart transplantations were subsequently performed worldwide in 1968. Medical staff used similar rhetoric to address the unique challenges they faced: ‘To become socially acceptable and to encourage organ donation, transplant surgery had to incorporate the dual discourses of the “gift of life” and “spare-part surgery”: the heart was the “ultimate” gift of life, yet just a replaceable pump’ (Nathoo, 2009: 183). Heart donations were portrayed as valuable, with the heart elevated to a life-giving and life-saving organ. Donors were depicted as ultimate altruistic benefactors. Simultaneously, the procedure was presented as a simple replacement of a mechanical body part, aiming to eliminate any doubts potential recipients might have and reinforce faith in transplantation medicine. This ‘ideological disjunction’ (Sharp, 2006: 14) became part of clinical practice in general. A dual process of depersonalization and personalization of organs emerged:

Whereas transplant recipients are encouraged by hospital staff to depersonalize their organs and speak of them in terms that can sometimes even approximate car repair, procurement staff regularly tell donor kin that transplantation enables the donor’s essence to persist in others who are thereby offered a second chance at life. (Sharp, 2006: 14)

### The transplantation and debate about David Bennett

Regarding the first xenotransplantation of a transgenic pig heart into a human in 2022, there are parallels to 1967. State actors, biotechnology companies, researchers, and physicians involved in xenotransplantation have a significant interest in its success and its integration into clinical routine as soon as possible. Consequently, there was a significant effort to portray the first xenotransplantation using a transgenic pig organ in a positive light, declaring it a medical success and scientific progress. While the transplantation was indeed portrayed positively in the media, it was not sensationalized to the extent it was in the 1960s. However, it also did not receive a great deal of attention from the public. Opinions expressed in the commentary sections of articles, such as those in the *Washington Post*, echoed those from 55 years ago: some voices applauded the procedure as an incredible feat, while others labelled it as ‘wrong’. Some also expressed the view that animals should not be killed for the benefit of humans, while others simply left humoristic comments (see Table 1).^2^

**Table 1.** Selected comments in the *Washington Post* following the report of the xenotransplantation (Pietsch, 2022).

Comments in favour of xenotransplantation	Comments against xenotransplantation	Humoristic/satirical comments
‘This is timely for that patient, and amazing during a pandemic’.	‘This is wrong. No human is worth more than an innocent animal’.	‘Yeah, but will it help him to more easily find truffles?’
‘This is a tremendous feat’.	‘This is truly a leap of faith’.	‘I hope this pig heart will be bacon him better….’
‘A truly remarkable achievement! Congratulations to the UMMC team, and best wishes for a full recovery to Mr. Bennett’.	‘So we humans have reached the point where no animal exists except to serve us. It’s not enough that they feed us. Now they have to give us their organs’.	‘They actually sought out a Republican patient to minimize the chance of rejection’.
‘Best of luck for a successful recovery; and hurray for the science progress!’	‘This is sick for so many reasons’.	‘I hope this pig heart will be bacon him better….’
‘Science has truly outrun science fiction when it comes to CRISPR’.	‘Another step down the SciFi road of genetically altered animals serving humans. Next stop, Planet of the Apes.’	Some pigs are more equal than others.
‘what astounding feats of science! I hope Mr Bennett makes a full recovery and that other patients all around the world may gain another chance at life!’	‘“If God intended man to fly He would have given him wings.” Actually, considering the benefit to mankind, and the number of pigs slaughtered for food on an annualized basis, genetically modified pigs could be raised for food and their hearts transplanted into cardiac patients, too. But the Bible says “No”’.	‘I have to ask: if he eats a ham sandwich is he considered to be a “cannibal”??:-)’
‘This is a fascinating development. [. . .] Gene editing has the potential to offer us great therapies. . .’	‘Oh wow. . . we are not even supposed to eat pig let have a part of one transplanted in us’.	
‘As someone whose been told I am headed for a heart transplant I am thrilled at this development’.	‘Our anthropocentrism has no limits.:(’	

UMMC: University of Maryland Medical Center; CRISPR: clustered regularly interspaced short palindromic repeats.

The public depiction of David Bennett paralleled that of Louis Washansky: an everyday person whom people could relate to, a family man who watched the Super Bowl from his hospital bed. However, public opinion rapidly changed after the media discovered a flaw in Bennett’s past. Just 2 days after the article about the successful transplantation was published in the *Washington Post* on 11 January, it was followed up by news of David Bennett’s criminal record. In 1988, Bennett had stabbed another man, Edward Shumaker, seven times, leaving him paralysed and requiring a wheelchair for the rest of his life. Bennett was sentenced to 10 years in prison but was released after six. The article focused on the perspective of Shumaker’s dependents and included a picture of Shumaker, bedridden, who passed away in 2007. Neither Shumaker nor his family ever received the monetary reparations that Bennett was ordered to pay (Johnson and Wan, 2022).

While matters of social justice were already invoked in 1967, this time the discussion was fuelled by news of Bennett’s past felony, shifting the direction of the debate. The initial article of the *Washington Post* generated 419 comments (the comments section was closed after 1 month), while the follow-up article about Bennett’s past garnered 3003 comments, more than seven times the response.

One may wonder why a presumed sensational scientific and medical wonder, an achievement successfully performed for the first time in the world, received less attention than a discussion on second chances, which had occurred many times before. One reason could be that the public readership was not fully aware of the surgery’s significance. This could be attributed to a deficit model, blaming an uninformed public or failed science communication. Indeed, who can claim to be ahead of ongoing medical advancements? In fact, the initial newspaper articles reporting on cardiac xenotransplantation, such as those in the *Washington Post* and the *New York Times*, provided relatively objective accounts of the procedure. While both articles referred to a ‘breakthrough’, they refrained from using sensational language and emphasized that there is still a long way to go before animal organ transplantation becomes a standard medical procedure.

However, the transplantation of a pig heart into a human should, naïvely speaking, elicit some reaction. Particularly for those unfamiliar with the state of medical and xenotransplantation advancements, this should evoke some kind of emotional response. The concept of xenotransplantation, when encountered for the first time, has been associated with a certain intuition, commonly referred to as the ‘yuck’ factor (Haddow, 2021).

As it may seem, medical sensations and technical progress do not always trigger reactions in the way one might expect. This may come as no surprise to social psychologists. For instance, Jonathan Haidt (2012) has identified five moral foundations that tend to evoke emotional responses among people. Scientific/technical progress is not one of them; however, fairness is among those foundations and can spark debates due to different conceptions of fairness, as evident in the ongoing discussion.^3^ Opinions vary depending on whether one believes in the state’s legal and justice system.

Some believe that David Bennett has paid his debt by serving his sentence, while others argue that the victim and his family have not received proper compensation and have been treated unfairly. For the latter group, justice should align with the principle of ‘an eye for an eye’. This raises the question of whether individuals deserve a ‘second chance’, as highlighted in the *Washington Post*’s headline (Johnson and Wan, 2022), which delves into the authentic capacity for remorse and personal transformation. Metaphorical language in the comments reflects these themes, considering the cultural significance attached to pigs and hearts. One *Washington Post* reader observes that the transplant recipient ‘already had the heart of a pig’, emphasizing Bennett’s perceived malicious nature (see Table 2). Twitter users have also noted that Bennett seems to have had a ‘change of heart’, employing irony or cynicism to highlight the possibility of becoming a different person through some form of catharsis.^4^ Meanwhile, some readers criticize the newspapers for publishing such supposedly provocative articles to incite such reactions.

**Table 2.** Selected comments in the *Washington Post* following the report of David Bennett’s criminal record (Johnson and Wan, 2022).

Comments identifying social justice	Comments identifying social injustice
‘He did his time. Whether you agree with the verdict or not there is no reason to deny him this experimental procedure. What is it with people who want others to continue to pay for their crimes long after they have served their sentence’.	‘Now it makes sense, He was a good candidate for the experiment because he already had the heart of a pig. Maybe the pig would be more deserving of his heart’.
‘We don’t have to forgive, but we do have to live within the boundaries of a civil society. He was tried, convicted, and did time for his crime. In a civil society he has paid his debt. The retributive sentiments on display here do not reflect a civil society but an eye for an eye culture’.	‘But Bennett literally didn’t pay. The court ordered him to make restitution to his victim and he thumbed his nose at the ruling. Just as he thumbed his nose at the medical requirements to do such difficult things as . . . showing up for appointments’.
‘Each of us is better than our worst moment, and worse than our best. If the criterion for receiving a transplant were having been a saint, there would be more than enough organs to go around and plenty left over’.	‘He can never repay his debt. The family will never recover, his victim will never have a life. There is no such thing as “Paying your debt to society” even for minor crimes. All of us suffer’.
‘Doctors are not supposed to treat people based on whether they are likable or not’.	‘Why was this monster chosen? He is clearly a cruel, vicious piece of garbage’.
‘I’m sorry that happened, but believe it or not, people can change. It was a horrible thing, but he served his time’.	‘Should have let the pig live and the violent ex-con die’.
‘How is this relevant to the medical procedure? This is yellow journalism at its finest, real National Inquirer stuff. WAPO likes to cause controversy, it seems to be their mantra now to try to stir things up and create conflicts, yet they like to trumpet their paper as ‘enlightened’ and taking the moral high ground. Digging up someone’s past like this – someone who is struggling to simply stay alive – is disgusting’.	‘A story made for Hollywood for sure. However, being the recipient of a pig heart as a some sort of ‘pioneer’ does not mean much, given his alternative – death. This will never make up for his brutal attack. But it is ‘something’ that may inspire a greater investment in xenotransplant research. And hopefully more worthy recipients will benefit in the future’.

WAPO: Washington Post.

## 3. Publics then and now

One may get the impression that not much has changed, except that in the past, readers expressed their opinions in the letters section of newspapers, while today, opinions are expressed in the commentary sections online or on social media. However, this serves as a reminder that public opinion is manipulated to the same extent as it was 50 years ago.

Two main aspects have changed. First, there is a specific point related to transplantation practice, namely the donors involved. Second, there are changes in the governance practices associated with biotechnology research and marketing. As a result, the public as an audience for newspaper and media reports no longer plays the same role as in the past. In pre-emptive biopolitics, attention is directed towards a different, engineerable form of the public.

### Change of paradoxes

The paradox associated with cardiac allotransplantation, where the heart is seen as the greatest gift one can give and, at the same time, a simple mechanical device that can be easily transferred, has been replaced by another paradox in the case of xenotransplantation.

In cardiac allotransplantation, the focus is on the donors, making the ‘gift of life’ meaningful and valuable. This was particularly the case in the 1960s:

Negotiating the role of the public was central. The notion of ‘the public’ was mobilized and utilized in myriad ways by different parties. Concurrently, ‘the public’ constituted patients – heart disease sufferers, organ recipients, potential organ donors, and those giving consent to organ donation, taxpayers and users of the NHS – and also media consumers. (Nathoo, 2009: 186)

Tensions in the medicine/science-media relationship may have remained the same, but the role of the public as a media audience has changed. In xenotransplantation, the appeal is not to the donor, as ‘donors’ can be farmed, but to individuals as potential recipients and beneficiaries of heart transplantation. This appeal overrides ethical considerations regarding animal welfare. The addressed audience does not need to be personally touched or affected as potential donors; it is sufficient to accept something that individuals have little control over as individuals. In other words, individuals are not asked to do much but simply to tacitly accept it, as they do with many other things in the world.

The paradox related to the conveyed messages has changed. One aspect remains the same, which is the feasibility of transplantation. The heart is viewed simply as a mechanical pump, disregarding species boundaries. It should be seen in a neutral and objective way. Just as little as the recipient should be concerned about the identity of the donor in allotransplantation, one should not be concerned about the identity of the organ source in xenotransplantation. While emphasizing the similarity between pigs and humans to make the transplantation seem insignificant, their differences in qualities such as reason, intellect, consciousness, awareness of harm and ability to feel pain are highlighted to underscore the qualitative difference and gap between species, affirming the superior worth, value, right to existence and right to survive vis-à-vis pigs. The fact that humans consume pigs makes this fact intuitively understandable. This ‘xenotransplantation paradox’ (Haddow, 2021), inherent in cross-species transplantation from animal organs to humans, has been observed by various social scholars studying xenotransplantation (e.g. Sharp, 2011 and in particular detail Cook, 2006). Besides, research has shown that xenotransplantation can cause disturbances regarding one’s (human) identity and self-image (Lundin, 2002).

### Biotechnology in times of pre-emptive biopolitics

However, the nature of biotechnology and the societal regime of science and technology have changed quite dramatically, and with it, the effort put into producing the kind of public sought. Also, allotransplantation does not presuppose a whole biotechnological industry of producing and farming donor animals. So, in order to understand the type of engagement with the public that biotechnology such as xenotransplantation participates in, we need to understand the mode of biopolitics in place.

In the 1990s, scientists in the United States worked on transplanting bone marrow from baboons to AIDS patients in order to improve immune responses to HIV. Fearing infectious diseases, particularly because non-human primates were believed to be the original carriers of HIV, the FDA put a ban on transplantations from non-human primates but remained permissive on xenotransplantation otherwise. In 1997, the transferability of porcine endogenous retrovirus (PERV) to human cells was proven. Consequently, the FDA and the US Public Health Services required stricter regulation guidelines for xenotransplantation research, yet they were otherwise supportive of xenotransplantation research as a means of alleviating the shortage of donor organs. The overall goal was to make xenotransplantation possible but to prevent interspecies contagions from occurring. ‘The *idea* of xenotransplantation involves a quintessentially liberal approach to borders and value: xenotransplantation seeks to circulate value across traditional boundaries between species’, Ray Carr (2022: 121) concludes. This reflects the widely held – at least in virology and public health – ‘view of an ecological body in which species, humans included, are partially permeable to flows of micro-organisms from within and without’ (Carr, 2022: 128).

The difference between this kind of biopolitics in contrast to the original form that Michel Foucault envisioned is as follows:

Foucault outlined security apparatuses that dealt with risks of a calculable probability occurring among populations and within a series of events, by redistributing and normalizing risks. New security apparatuses [. . .] are fundamentally speculative; they target uncertain and incalculable future emergence and emergencies with a range of imaginative, pre-emptive, and future invocative tools. [. . .] Contemporary public health strategies have responded to speculative apprehension of looming disease threats by increasing surveillance, innovation, and circulation of biological fragments, to pre-empt and prepare for fundamentally unpredictable pandemics. (Carr, 2022: 121–122)

The imagination of a future catastrophic spreading of PERV or other viruses has called for pre-emptive measures. This is partly based on the precautionary principle calling for a moratorium on xenotransplantation research, as it has been put in place in some countries for a limited period of time. However, what prevailed is the pre-emption rationale of investing in biotechnology to find a fix (and thereby also in speculative capital turnout).

Following the emphasis of ‘pre-emption’ (Carr, 2022; Cooper, 2006) within the ‘contemporary biopolitics of preparedness and resilience’ (Carr, 2022: 118), I will speak of ‘pre-emptive biopolitics’.

It should be clear that this pre-emptive biopolitics (as biopolitics before) is not solely a matter restricted to the government, unlike the previous mode of sovereign apparatuses. As such, it does not simply involve the passing of laws or the installation of prohibitive rules. Instead, it represents a form of governance that is intended to enable individuals and institutional actors to act ‘freely’ in accordance with the prevailing doctrine, which, in this case, aligns with the understanding of the ecological body and emphasizes pre-emptive measures rather than prohibitive ones.

An indicative example of this development is a debate that unfolded in Nature Biotechnology. It began with an opinion piece by legal scholars identifying various areas in xenotransplantation that lacked regulation. This prompted immediate responses from the FDA and representatives of the International Xenotransplantation Association (IXA). The main critique, and even the only one (except for one specific point raised by the FDA), was primarily formal in nature and not directly related to the content of the target article. Particularly in the IXA article, the emphasis was on the fact that regulatory responsibilities had been assumed by the respective organizations, highlighting the series of meetings held and the guidelines or communiques that were issued. However, there was no specification of what had been regulated or how it addressed the identified gaps in the target article. The focus was primarily on emphasizing the fact that regulation was being taken care of.^5^

## 4. Publics and public debate

‘The public’ denotes an ephemeral term that is difficult to define satisfactorily. However, what I am interested in is the relationship between science and the public, particularly when science projects aim to gain favourable public opinion. In this regard, citizen participation has become an increasingly popular approach. In order to approximate the meaning of ‘public’ or the specific type of public being referred to in this changing context, let’s briefly explore the ways in which the public is conceptualized in the literature.

### Typifications of the public

Braun and Schultz (2009) have identified four types of public: the general public, the pure public, the affected public and the partisan public. The general public is typically assessed through opinion polls and surveys. The results are considered ‘raw and potentially unreliable’ and require experts to properly interpret them (Braun and Schultz, 2009: 409). In contrast to the general public, the pure public does not represent existing opinions and attitudes but aims to cultivate transformed and refined opinions. Through educational processes, citizens and laypeople are empowered to become knowledgeable and well-informed individuals. This transformation is achieved through participation formats such as citizen/consensus conferences and citizen juries. On the other hand, the affected public consists of individuals who possess firsthand knowledge and immediate emotional involvement in the issue at hand, such as patients suffering from a particular condition. The affected public not only provides valuable knowledge but also offers ‘emotional education’ (Braun and Schultz, 2009: 411). Consultative panels, for example, seek to incorporate the perspective of the affected public alongside traditional expert opinions within decision-making bodies. Finally, there is the partisan public, which represents the opinions of interest groups, stakeholder organizations and lobbying bodies. This type of public is considered inauthentic compared to the others. Nonetheless, stakeholder consultations are conducted to understand their viewpoints and perspectives.

While public governance, whether conducted by a government or the biotech industry (often it is impractical to identify a single actor; instead, one should consider the dominant biopolitics or zeitgeist), aspires to shape engineered publics, preferably those considered ‘authentic’, another characteristic of these engineered publics is their subjective nature. Citizens involved in these publics, prompted by the (neoliberal) offer or imperative of citizen participation, embrace the opportunity, take on the responsibility, and fulfil the role of ‘doing public’ (Michael, 2009).

Mike Michael (2009) introduces two rhetorical categories of publics: Publics-in-General and Publics-in-Particular. Publics-in-Particular are ‘those publics that have an identifiable stake in particular scientific or technological issues or controversies’ and ‘can be associated with specific scientific projects, programs of research or technoscientific enterprises, are attached to recognizable “interests”, and enact particular alliances with other actors’ (Michael, 2009: 623). In these terms, the four publics identified earlier by Braun and Schultz can all be considered as Publics-in-Particular. Publics-in-General, on the other hand, are viewed as an undifferentiated whole characterized by their opposition to science.

There is no public outside or beyond the ones being constructed (e.g. public opinion surveys, consensus conferences). However, these constructed publics do not necessarily reflect the desired outcome, which is public acceptance and somewhat elusive support from everyone. Nonetheless, these results are often used to demonstrate ‘public acceptance’. As an example, a Public-in-Particular, such as a citizen conference on xenotransplantation, can be used to represent a Public-in-General by claiming ‘public acceptance’ of xenotransplantation (Alberio and Wolf, 2021). When examining the public created around the topic of xenotransplantation, Michael and Brown (2004) identify the public with a pro-stand, such as patient groups that legitimize the use of pig organs by referring to the commonplace consumption of pork and the public with a con-stand, who draw parallels between laboratory animals and pets, advocating equal treatment for both.

### Citizen participation

For specific biotechnological research endeavours like xenotransplantation, the goal is not to reach every individual, although pre-emptive biopolitics also subtly affect individuals. Instead, the aim is to achieve something akin to ‘public acceptance’. However, the path to achieving public acceptance is not clear. There is no public vote on specific biotechnologies, and in many cases, it would not be desirable, particularly in xenotransplantation where there is a widespread aversion, often associated with the ‘yuck’-factor. In addition, the official endorsement of democratic representatives, which is considered the legitimate voice of the public, has been questioned in the era of proclaimed post-democracy. In fact, democratic governments are starting to initiate citizen council projects themselves. As a result, manageable-sized publics are engineered, such as citizen conferences (Bogner, 2011). These conferences tend to produce results that align with official law-making. However, they face challenges such as reflecting the public discussion and framing of the topics discussed, while the organizing entities’ external conditions and engineering also play a role.

At least four points of critique regarding citizen participation can be identified (Kögel, 2021): its (a)political nature as a social technology, the lack of representativeness and legitimacy, the enactment of participation, and its limited impact.

(A)political social technology: Through citizen participation, politics aims to compensate for its increasing legitimacy deficit and gain citizens’ confidence. By implementing ‘engineered citizenship’, citizens are transformed into a resource for optimizing decision-making through innovative participatory formats (Münte, 2017: 176). Consequently, rather than improving democratic practice, citizen participation can be seen as a technocratic regime (Rayner, 2003) that shifts responsibility from state officials/politicians to citizens (Maasen and Kaiser, 2007). As a result, we witness a ‘depoliticization by participation’ (Münte, 2017: 176). While intended to foster political decision-making, its imitation has a counterproductive political impact.
Lack of representativeness and legitimacy: Citizen participation can pose a threat to democracy as it perpetuates social inequality. This is due to the lack of representativeness among its participants. Neither the economically disadvantaged class, partly due to their disillusionment with politics (Jörke, 2011), nor the elite class (Selle, 2011) engage in participation projects. Consequently, there is a strong bias towards members of the educated middle class. Moreover, citizen participation lacks legitimacy as it is neither initiated through popular election nor based on constitutional procedures (Maasen and Kaiser, 2007).
Enactment of participation: Citizen participation often appears as a socially engineered practice that suggests and imitates decision-making procedures but lacks binding authority. Participation then becomes a form of ‘particitainment’, where participation is pursued for its own sake (Selle, 2011). In addition, participation formats are considered as ‘laboratory experiments’ in nature, as the conditions are controlled by (social) scientists (or participation experts) (Bogner, 2010). These processes tend to steer participants towards a dominant opinion, known as ‘mainstreaming’ (Bogner, 2010), ultimately aiming to reach a consensus (Martinsen, 2001). Steve Rayner (2003: 169) stated that citizen participation was ‘consensus seeking with respect to both knowledge and values and, as such, it is depoliticizing’.
Limited impact: Since citizen participation is not deeply embedded in democratic or political institutions, its formats often end up being either ‘little more than focus groups’ or serving as legitimating devices for pre-determined policies (Pateman, 2012: 9). In general, Goodin and Dryzek (2006) find little evidence of direct relationships between citizen participation and political decisions.

In the specific case of xenotransplantation, the critique is that public consultation formats primarily serve the purpose of acquiring social legitimization (Sobbrio and Jorqui, 2014). Erich Griessler et al. (2012) examined citizen participation projects on xenotransplantation in terms of their impact on policymaking. While they could not identify any direct political influence, referred to as ‘first generation impact’, they observed ‘second and third generation’ consequences, which involve reception by civil society (news reporting, public debate, politicization of stakeholder groups, discourse category building). In Switzerland and the Netherlands, governmental institutions organized citizen participation initiatives on xenotransplantation. However, both countries passed bills regulating xenotransplantation before the respective participation formats had finalized their conclusions (Griessler et al., 2012). Imitating democratic processes without wielding actual power can be considered as ‘simulative democracy’ (Blühdorn, 2020).

### Xenotransplantation and public consultation

Taking a step back, ‘xenotransplantation has found itself at the crossroad of different emerging regulatory principles and practices, becoming the experimentation terrain for new ways of framing and practicing democracy in science-based policies, innovating the governance of science, and protecting citizens from technological risks’ (Sobbrio and Jorqui, 2014: 524). Xenotransplantation may have been a contingent choice to signify a watershed event in scientifically engineered public participation. Nevertheless, it became the focal issue that sparked a wave of public consultation formats, leading to the recognition that ‘the public’, previously treated as a singular term, exists in plural (Sobbrio and Jorqui, 2014). Simultaneously, xenotransplantation was the first medical procedure that supposedly required social recognition and the first subject that invoked the application of the precautionary principle beyond environmental concerns (Sobbrio and Jorqui, 2014). According to Sobbrio and Jorqui (2014), the relationship between the precautionary principle and xenotransplantation was initially conceptualized by the Nuffield Council in 1996. Consequently, the precautionary principle became established within biomedicine and public health as a viable approach, and xenotransplantation became a topic for discussion in public consultation formats. Furthermore, changes occurred in defining and qualifying ‘the public’. Sobbrio and Jorqui (2014) outline the history of ‘the public’ in xenotransplantation research. In the 1990s, studies were initiated to assess public opinion on xenotransplantation, primarily through surveys. While most of these studies reported opinions in favour of xenotransplantation, sceptical assessments in Australia led to conflicting research outputs and created a ‘war of numbers [. . .] and local attitudes’ (Sobbrio and Jorqui, 2014: 527). During the same period, quantitative studies, primarily surveys, were used to promote the issue of xenotransplantation, while qualitative studies were considered inadequate. In the 2000s, citizen participation emerged as a relevant tool in science and technology communication, particularly in a changing political context. In contrast to descriptive survey studies, participation projects were initiated, promoting a ‘normative understanding of the role of the citizen’, where participating citizens were expected to act as ‘responsible co-policy makers’ and hence engage in ‘scientific citizenship’ (Sobbrio and Jorqui, 2014: 528). Unlike surveys that assess the public’s ‘raw opinion’, deliberative formats aim to obtain a ‘refined opinion’ that emerges after proper information sharing, expert input, and argumentative procedures among participants (Fishkin, 2009). These procedures have been implemented in Canada (Einsiedel, 2002), Australia (Cook, 2011), New Zealand (Thomas, 2007), Switzerland (Griessler, 2011), the Netherlands (Versteeg and Loeber, 2011), and Germany (Kögel and Marckmann, 2020).

In the case of xenotransplantation, deliberative formats appear particularly valuable due to its affective dimension. Unlike other technologies where controversy or opposition may emerge only after gaining information, xenotransplantation can evoke strong emotions when people encounter it for the first time. Intuitive responses may include disgust or a feeling that something must be wrong with it. These opinions can change during the course of the ‘refining’ process in deliberative projects (Kögel and Marckmann, 2021).

It is crucial to note that ‘publics do not exist per se, without public consultations, surveys, public conferences, etc. All of them constitute participation methods to facilitate the incorporation of their voices and perspectives into decision-making’ (Sobbrio and Jorqui, 2014: 530). Through public consultation on xenotransplantation, various ‘publics’ have been formed, comprising different population groups such as medical professionals, nurses, students, patients, or the ‘general public’. Usually, the term ‘general public’ refers to a representative sample of the population when no specific group is addressed.

The fervent call to engage the public and gain their acceptance of xenotransplantation may seem surprising, at least in part. Logically, ethicists and social scientists emphasize the need to include everyone in decisions on matters that affect them for the sake of democracy. However, most Western countries have a representative parliamentary form of democracy. Therefore, stakeholders of emerging biotechnologies primarily require the consent of legislation or appropriate legal regulations. This can be easily achieved through the connection of the pharmaceutical industry to politics, as they also have a stake in the matter once they are involved in a particular technology. Typically, there is no need to engage the public, and it has rarely been done in other biomedical projects. So why do legal and medical scholars, as well as scientists, also call for public engagement? Perhaps it is to encourage the involvement of pharmaceutical companies through a positive social reputation or public pressure. Partly, there also appears to be anxiety about causing social protests, as seen in the past with issues like genetically modified organisms (GMO) or bovine spongiform encephalopathy (BSE) in the UK. In the case of xenotransplantation, significant effort is made to ensure public agreement or at least tolerance. As Irwin and Michael (2003: 145) observed,

Thus, to develop a policy-making strategy on animal experimentation (i.e., to establish institutional bodies that can address the issues around animal experimentation in order to develop policy), one must choose which portion of the public to consult and/or invite into the process of discussion and argumentation.

This is because ‘public legitimacy is not just a democratic virtue; public acceptance is needed for research to develop and thrive’ (Andreasen and Hoeyer, 2009: 543). In general, Irwin and Michael (2003: 91) state that ‘for science policy, the public is particularly important because it embodies certain values (such as those concerning the environment or animal rights or animal welfare) that are seen as necessary for the policy-making process’.

### The nature of unmediated publics

The public under consideration here, the debate on social media and in commentary sections, is not an engineered form of the public, nor is it a semantic used to justify or legitimize a point or matter (except for the argument made in this article). The ensuing debate can be viewed as peculiar from various perspectives. First, one might have expected a discussion centred around the threat of xenozoonoses, considering the heightened sensitivity towards that topic due to dealing with the SARS-CoV-2 pandemic for a period of 2 years at that time. However, what emerged was not a debate on public health or even medical-related issues but rather a controversy regarding social justice. The unfolding debate, which revolved around the discussion of social justice, was hardly the publicity the xenotransplantation alliance had hoped for. They had hoped for reactions that would celebrate the procedure as a landmark success, or even demands for increased support and development of xenotransplantation research. Defending their doing, the official response of the University of Maryland Medical Center to the unfolding debate, as voiced in the *New York Times* (Rabin, 2022b), is as follows:

It is the solemn obligation of any hospital or healthcare organization to provide lifesaving care to every patient who comes through their doors based on their medical needs. [. . .] Any other standard of care would set a dangerous precedent and would violate the ethical and moral values that underpin the obligation physicians and caregivers have to all patients in their care.

The same article featured the opinion of the Organ Procurement and Transplantation Network, responsible for the transplant list in the United States, which stated that ‘punitive attitudes that completely exclude those convicted of crimes from receiving medical treatment, including an organ transplant, are not ethically legitimate’.

These statements simply align with the structure of society in terms of its functional differentiation. I understand the functionally differentiated society, following Niklas Luhmann (2012), as consisting of various functional systems (such as politics, law, the economy, religion, the arts, science, education, sports, mass media), each comprising its respective logic/binary codes, mode of communication/symbolic generalization, self-referential regulatory autonomy, and operative closure (Stichweh, 2013). What occurs within the legal system (e.g. the conviction of David Bennett) does not directly translate into consequences within the medical system (e.g. the treatment of David Bennett). However, there is always some integration among the different subsystems (Luhmann, 2012). The normative realm cuts across these subsystems, as any interaction or communication can have normative implications. One could argue that society’s function is to prevent the normative standards of one system from influencing the others. As we have seen and argued, David Bennett’s criminal record is irrelevant when discussing medical ethics. Medicine, or the healthcare sector, simply has no interest in the personal background of its patients or customers. In the eyes of the law, once you have served your sentence (unless you commit another offence), you are free and free of charges. However, since the normative dimension cuts across these spheres (and because people generally do not concern themselves with society’s functional differentiation), there is a temptation to discuss these ethical matters as if no functional boundaries existed. This is why such discussions exist and why stakeholders in xenotransplantation are interested in public acceptance of their technology.

In addition, we have observed instances of subsystem integration when looking at particular individuals’ culmination points. An individual may be a customer or seller in the economic realm, a taxpayer, voter, citizen and/or politician in the political realm, a legal subject or lawyer in the legal realm, a healthcare recipient or doctor in the medical realm, and so on. However, there can be a tendency observed across these systems. This phenomenon has been described as the Matthew principle or Matthew effect. Those who are gifted or hold high ranks in one sphere tend to have the same standing in others. The wealthy often possess political power, enjoy more rights, win legal cases more frequently, and have better health, while the poor, powerless and sick face the opposite. Being a convicted felon may also lead to an inferior health status. In fact, David Bennett’s health was not optimal. Yet, the reasons behind this were social: he was non-compliant with his doctor’s prescriptions and smoking and alcohol left their mark on his body.

These factors are not subject to interference by society through political or legal measures. They are meant to maintain functional differentiation, which involves officially ignoring interferences. That is why David Bennett became a deserving recipient of a xenograft and hence, depending on one’s assessment of the life expectancy of xenotransplantation at that stage, a reasonable candidate for a ‘second chance’. That is also why science cannot directly influence politics or the media. As seen in the case of David Bennett, the discussion revolved around whether he deserved the transplant or not, rather than acknowledging the potential of a new remedy for organ shortage.

On a short theoretical note: Each function system has developed its particular performance roles as well as complementary lay roles (such as the patient, the customer, the voter) (Stichweh, 2021). The ‘citizen’ as the layperson who volunteers as a responsible participant can either be seen as taking up this complementary role within the system of science or denotes a specification or even secondary performance role within the political system. This depends on the outset of the participation format, either as citizen science or participatory research (aiming at knowledge production/truth-finding), or as a political deliberation/participation project (directed at the process of collectively binding decision-making).

In this context, it becomes evident that translational work is needed to convey the logic and content of one functional system to another, as well as between professionals and laypeople within each system. This is why medical experts and scientists are brought together with social researchers and participation professionals (who have a mediating role), along with citizens representing the lay public.

It is worthwhile to note also that the science system communicates through popularization, starting from one colleague having to break things down to explain to another colleague, up to explaining it to public audiences (Stichweh, 2003).

Regarding the unmediated public, the media acts as a medium that conveys information, stories and news but does not mediate people’s opinions in a way that encourages engagement with each other, other opinions and arguments to reach a shared opinion, statement or consensus. There is no mediation aimed at refining opinions. Because this kind of public is not engineered or directed, its outcome is completely unpredictable and can render unwanted results.

## 5. Conclusion

Where privacy is manufactured in its most conspicuous form – most visibly in self-promotion on social media, not confined to, but particularly conspicuous in influencers’ and other professional content creators’ – it is seen as giving an account of the real, that is, ‘authentic’, world (Bauer, 2018). Authenticity here is not based on the truthfulness of the account, but on the direct, unmediated transfer of personal information; as for the audience, the streamer/content producer is talking to them directly, not to the camera. Accordingly, in terms of power, the privacy model of surveillance, as envisioned by Foucault, can be seen as being replaced by the model of ‘capture’ of Philip Agre (1994).

Consequently, what then counts as an authentic public is the engineered public displayed by opinions gained through some deliberative or participatory procedure representing the ‘authentic’ perspective of ordinary citizens. What people write in commentary columns, by this logic, can by all means be neglected. Yet, lessons from the past may serve as a caveat for our times: ‘The extensive and unmanageable media coverage had major negative implications for heart transplantation, other transplant programmes, and for the wider medical community’ (Nathoo, 2009: 184). A spate of unsuccessful heart transplantations worldwide in 1968 led to a decline in its acceptance among the public and medical peers, a fear that also reigns today (Chaban et al., 2022).

Unmediated publics, such as the debate following the xenotransplantation of David Bennett and the revelation of his past, take their own chaotic trajectory. This can be seen as an immediate public dynamic and a democratic accomplishment. At the same time, it can be argued that mediated or engineered publics are necessary simply to mitigate the translational work among various subsystems in a functionally differentiated society and to keep democracy alive and up to the task of increasing complexity of society at large, particularly because of the increasing complexity of the knowledge produced within these subsystems and the increasing difficulty of negotiating between them. Citizen participation serves as a means of fostering mutual understanding and interaction between science, the public and politics. The implementation of these forms of citizen participation will determine their effectiveness. However, they should not be regarded as ‘the public’.

## Notes

1. For a discussion of the ‘half-identity’ in xenotransplantation, see Cook and Osbalidston, 2010.

2. As the *New York Times* and the *Washington Post* were the first newspapers to break the news on David Bennett’s story, I took a look at the respective comment sections (the one for the *New York Times* on Twitter). For illustrative purposes, some comments from the *Washington Post* are listed. However, I did not conduct a systematic analysis. My interest lies not specifically in the content of the comments but in the observation that the debate transformed into a discussion of social fairness.

3. There is also the foundation of ‘sanctity’, which Haidt identifies as particularly applicable to biomedical issues. According to this concept, intrusion into the body can be condemned. However, Haidt does not attribute the same significance across the political spectrum to sanctity as he does to fairness.

4. For the Twitter comments, see https://twitter.com/nytimes/status/1481732839976624143 (accessed 8 December 2023).

5. The IXA, nevertheless, must be regarded as a powerful institution. For example, it managed to clamp down on xenotransplantation research in Mexico, which was not performed in line with its standards, an intervention that could be seen as ‘medical imperialism’ (Cook et al., 2011).

## References

[bibr1-09636625241232098] Agre PE (1994) Surveillance and capture: Two models of privacy. The Information Society 10(2): 101–127.

[bibr2-09636625241232098] Alberio R Wolf E (2021) 25th Anniversary of cloning by somatic-cell nuclear transfer: Nuclear transfer and the development of genetically modified/gene edited livestock. Reproduction 162(1): F59–F68. 10.1530/REP-21-0078 PMC8240728 34096507

[bibr3-09636625241232098] Andreasen M Hoeyer K (2009) DNA patents and the invisible citizen: The role of the general public in life science governance. SCRIPTed 6: 538–557.

[bibr4-09636625241232098] Bauer T (2018) Die Vereindeutigung der Welt: Über den Verlust an Mehrdeutigkeit und Vielfalt [The Unification of the World: On the Loss of Ambiguity and Diversity]. Stuttgart: Reclam.

[bibr5-09636625241232098] Blühdorn I (2020) The dialectic of democracy: Modernization, emancipation and the great regression. Democratization 27(3): 389–407.

[bibr6-09636625241232098] Bogner A (2010) Partizipation als Laborexperiment: Paradoxien der Laiendeliberation in Technikfragen [Participation as a Laboratory Experiment: Paradoxes of Deliberation on Technology Issues by Lay People]. Zeitschrift für Soziologie 39(2): 87–105.

[bibr7-09636625241232098] Bogner A (2011) The paradox of participation experiments. Science, Technology, & Human Values 37(5): 506–527.

[bibr8-09636625241232098] Braun K Schultz S (2009) ‘. . . a certain amount of engineering involved’: Constructing the public in participatory governance arrangements. Public Understanding of Science 19(4): 403–419. 10.1177/0963662509347814 20977180

[bibr9-09636625241232098] Carr R (2022) Species of Contagion: Animal-to-Human Transplantation in the Age of Emerging Infectious Disease. Singapore: Palgrave Macmillan.

[bibr10-09636625241232098] Chaban R Cooper DK Pierson III (2022) Pig heart and lung xenotransplantation: Present status. The Journal of Heart and Lung Transplantation 41(8): 1014–1022. 35659792 10.1016/j.healun.2022.04.010 PMC10124776

[bibr11-09636625241232098] Cook P (2006) Science stories: Selecting the source animal for xenotransplantation. In: Hall C Hopkinson C (eds) Social Change in the 21st Century 2006 Conference Proceedings. Brisbane, QLD, Australia: Queensland University of Technology, pp. 1–17.

[bibr12-09636625241232098] Cook PS (2011) What constitutes adequate public consultation? Xenotransplantation proceeds in Australia. Journal of Bioethical Inquiry 8(1): 67–70.

[bibr13-09636625241232098] Cook PS Kendall G Michael M Brown N (2011) The textures of globalization: Biopolitics and the closure of xenotourism. New Genetics and Society 30(1): 101–114.

[bibr14-09636625241232098] Cook PS Osbaldiston N (2010) Pigs hearts and human bodies: A cultural approach to Xenotransplantation. M/C Journal 13(5).

[bibr15-09636625241232098] Cooper M (2006) Pre-empting emergence: The biological turn in the war on terror. Theory, Culture & Society 23(4): 113–135.

[bibr16-09636625241232098] Douglas M (1976) Purity and Danger. London: Routledge and Keegan Paul.

[bibr17-09636625241232098] Einsiedel EF (2002) Assessing a controversial medical technology: Canadian public consultations on xenotransplantation. Public Understanding of Science 11(4): 315–331.

[bibr18-09636625241232098] Fishkin J (2009) When the People Speak: Deliberative Democracy and Public Consultation. Oxford: Oxford University Press.

[bibr19-09636625241232098] Goodin R Dryzek J (2006) Deliberative impacts: The macro-political uptake of mini-publics. Politics & Society 34: 219–244.

[bibr20-09636625241232098] Griessler E (2011) Xenotransplantation policy and participatory technology assessment in Switzerland. IHS Sociological Series, Working Paper 95. Available at: https://core.ac.uk/download/pdf/212121399.pdf (accessed 10 June 2023).

[bibr21-09636625241232098] Griessler E Biegelbauer P Hansen J Loeber A (2012) Citizen Participation in Decision-Making on Complex and Sensitive Issues? Experiences with Xenotransplantation. Austria: CIT-PART Consortium.

[bibr22-09636625241232098] Haddow G (2021) ‘Dirty pigs’ and the xenotransplantation paradox. Medical Humanities 47(4): 417–424. 34697231 10.1136/medhum-2021-012187 PMC8639932

[bibr23-09636625241232098] Haidt J (2012) The Righteous Mind: Why Good People Are Divided by Politics and Religion. New York, NY: Pantheon Books.

[bibr24-09636625241232098] Irwin A Michael M (2003) Science, Social Theory and Public Knowledge. Maidenhead: Open University Press.

[bibr25-09636625241232098] Johnson L Wan W (2022) The ethics of a second chance: Pig heart transplant recipient stabbed a man seven times years ago. The Washington Post. Available at: www.washingtonpost.com/dc-md-va/2022/01/13/pig-heart-transplant-stabbing-david-bennett/ (accessed 10 June 2023).

[bibr26-09636625241232098] Jörke D (2011) Bürgerbeteiligung in der Postdemokratie [Citizen Participation in Post-Democracy]. APuZ 1–2: 13–:18.

[bibr27-09636625241232098] Kögel J (2021) Zwischen Technikfolgenabschätzung und Demokratieprojekt: Herausforderungen von Bürgerbeteiligungsverfahren [Between Technology Assessment and Democracy Project: Challenges of Citizen Participation Procedures]. In: Kögel J Marckmann G (eds) Xenotransplantation als gesellschaftliche Herausforderung. Die Münchner Bürgerkonferenz: Hintergründe – Verfahren – Ergebnisse – Reflexionen [Xenotransplantation as a Societal Challenge. The Munich Citizens’ Conference: Backgrounds – Procedures – Results – Reflections]. Leiden: Mentis, pp. 21–46.

[bibr28-09636625241232098] Kögel J Marckmann G (2020) ‘Xenotransplantation challenges us as a society’: What well-informed citizens think about xenotransplantation. EMBO Reports 21(9): e50274. 32783261 10.15252/embr.202050274 PMC7507413

[bibr29-09636625241232098] Kögel J Marckmann G (eds) (2021) Xenotransplantation als gesellschaftliche Herausforderung. Die Münchner Bürgerkonferenz: Hintergründe – Verfahren – Ergebnisse – Reflexionen [Xenotransplantation as a Societal Challenge. The Munich Citizens’ Conference: Backgrounds – Procedures – Results – Reflections]. Leiden: Mentis.

[bibr30-09636625241232098] Kotz D (2022) University of Maryland School of Medicine Faculty Scientists and Clinicians perform historic first successful transplant of porcine heart into adult human with end-stage heart disease. Available at: www.medschool.umaryland.edu/news/2022/University-of-Maryland-School-of-Medicine-Faculty-Scientists-and-Clinicians-Perform-Historic-First-Successful-Transplant-of-Porcine-Heart-into-Adult-Human-with-End-Stage-Heart-Disease.html (accessed 10 June 2023).

[bibr31-09636625241232098] Luhmann N (2012) Theory of Society, vol. 1. Stanford: Stanford University Press.

[bibr32-09636625241232098] Lundin S (2002) Creating identity with biotechnology: The xenotransplanted body as the norm. Public Understand of Science 11(4): 333–345.

[bibr33-09636625241232098] Maasen S Kaiser M (2007) Vertrauen ist gut. Verantwortung ist besser. Die Herstellung von Verantwortlichkeit in der partizipativen Technikfolgenabschätzung [Trust is Good. Responsibility is Better. The Production of Accountability in Participatory Technology Assessment]. In: Porz R Rehmann-Sutter C Scully JL Zimmermann-Acklin M. (eds) Gekauftes Gewissen? Zur Rolle der Bioethik in Institutionen [Purchased Conscience? On the Role of Bioethics in Institutions]. Paderborn: Mentis, pp. 75–87.

[bibr34-09636625241232098] Martinsen R (2001) Ethikpolitik als mentale Steuerung der Technik: Zur Kultivierung des Gewissens im Diskurs [Ethics Policy as Mental Control of Technology: On Cultivating Conscience in Discourse]. In: Simonis G Martinsen R Saretzki T (eds) Politik und Technik [Politics and Technology]. Wiesbaden: VS Verlag für Sozialwissenschaften. pp. 499–525.

[bibr35-09636625241232098] Michael M (2009) Publics performing publics: Of PiGs, PiPs and politics. Public Understanding of Science 18(5): 617–631.

[bibr36-09636625241232098] Michael M Brown N (2004) The meat of the matter: Grasping and judging xenotransplantation. Public Understanding of Science 13(4): 379–397.

[bibr37-09636625241232098] Münte P (2017) Improving modern society: Governing science and technology by engineered participation. In: Paul R Mölders M Bora A Huber M Münte P (eds) Society, Regulation and Governances. New Modes of Shaping Social Change? Cheltenham: Edward Elgar Publishing, pp. 166–180.

[bibr38-09636625241232098] Nathoo A (2009) Hearts Exposed: Transplants and the Media in 1960S Britain. Basingstoke: Palgrave Macmillan.

[bibr39-09636625241232098] Pateman C (2012) Participatory democracy revisited. Perspectives on Politics 10(1): 7–19.

[bibr40-09636625241232098] Pietsch B (2022) In first surgery of its kind, Maryland man receives heart transplanted from genetically modified pig. The Washington Post. Available at: https://www.washingtonpost.com/science/2022/01/11/pig-heart-transplant-genetically-modified/ (accessed 10 June 2023).

[bibr41-09636625241232098] Rabin RC (2022a) In a first, man receives a heart from a genetically altered pig. The New York Times. Available at: https://www.nytimes.com/2022/01/10/health/heart-transplant-pig-bennett.html (accessed 10 June 2023).

[bibr42-09636625241232098] Rabin RC (2022b) Patient in groundbreaking heart transplant has a violent criminal record. The New York Times. Available at: https://www.nytimes.com/2022/01/13/health/pig-heart-transplant-bennett.html (accessed 10 June 2023).

[bibr43-09636625241232098] Rayner S (2003) Democracy in the age of assessment: Reflections on the roles of expertise and democracy in public-sector decision making. Science and Public Policy 30(3): 163–170.

[bibr44-09636625241232098] Selle K (2011) ‘Particitainment’ oder: Beteiligen wir uns zu Tode [‘Particitainment’ or: Are We Participating to Death]? pnd / Online 3: 1–19.

[bibr45-09636625241232098] Sharp LA (2006) Strange Harvest: Organ Transplants, Denatured Bodies, and the Transformed Self. Berkeley, CA: University of California Press.

[bibr46-09636625241232098] Sharp LA (2011) Monkey business: Interspecies longing and scientific prophecy in experimental xenotransplantation. Social Text 29(1(106)): 43–69.

[bibr47-09636625241232098] Sobbrio P Jorqui M (2014) An overview of the role of society and risk in xenotransplantation. Xenotransplantation 21(6): 523–532. 25040770 10.1111/xen.12120

[bibr48-09636625241232098] Stichweh R (2003) The multiple publics of science: Inclusion and popularization. Soziale Systeme 9(2): 210–220.

[bibr49-09636625241232098] Stichweh R (2013) The history and systematics of functional differentiation in sociology. In: Albert M Buzan B Zürn M (eds) Bringing Sociology to International Relations: World Politics as Differentiation Theory. Cambridge: Cambridge University Press, pp. 50–70.

[bibr50-09636625241232098] Stichweh R (2021) Individual and collective inclusion and exclusion in political systems. In: Ahlers AL Krichewsky DM Evelyn Stichweh R (eds) Democratic and Authoritarian Political Systems in 21st Century World Society. Bielefeld: Transcript, pp. 13–38.

[bibr51-09636625241232098] Thomas C (2007) Public dialogue and xenotransplantation. Medicine and Law 26: 801–815. 18284119

[bibr52-09636625241232098] Versteeg W Loeber A (2011) CIT-PART: Report Case Study Netherlands. Amsterdam: University of Amsterdam.

